# American Academy of Medicine

**Published:** 1888-12

**Authors:** 


### AMERICAN ACADEMY OF MEDICINE.

The twelfth annual meeting of the Academy occurred in New York, November 13 and 14.

After reading of the minutes of the preceding annual meeting, the deaths of Drs. C. R. Agnew and Howard Pinkney, of New York City; M. H. Bosland, of Pittsburgh, Pennsylvania; Theodore T. Wing, of Susquehanna, Pennsylvania; E. S. Dunster, Ann Arbor, Michigan, and Stephen B. Kieffer, of Carlisle, Pennsylvania, were announced.

A report of the standing committee on the requirements for preliminary education in the various medical colleges in the United

States and Canada was made through Dr. Leartus Connor, of Detroit, Michigan. It stated that in the United States there are 116 medical schools, of which it is claimed that 89 announce certain preliminary educational requirements which vary considerably. The report maintains that the most effective way of elevating the standard of requirements is by the united effort of the profession to secure definite preliminary education as an essential to the beginning of the study of medicine.

Dr. J. C. WILSON, of Philadelphia, Pennsylvania, read a paper on the "Causes and Prevention of the Opium Habit and Kindred Affections."

He divided the causes into three classes:

1. Example, as shown in case of those who become opium-eaters through the example of friends.

2. Suggestion, shown in those who acquire the habit through the reading of literature concerning it, or from familiarity with the drugs, as in case of physicians, students of medicine, druggists, and nurses.

3. Medical prescriptions.

It is an unfortunate fact that the greater number of the victims to the habitual use of opium become such through abuse of opiates originally prescribed for the relief of pain.

As measures for the prevention of such habits he suggested—

(*a*) The dissemination of proper knowledge of the methods by which the opium-habit is acquired, and the great dangers of this habit;

(*b*) A reasonable presentation of the facts in popular works upon hygiene; and,

(*c*) Proper protection on the part of physicians in prescribing narcotics.

He claimed that prescriptions for narcotics should not be renewed by druggists without the written order of the physician who had prescribed them, and that only in the most exceptional cases should the hypodermic syringe be prescribed for the patient's use himself. He maintained that existing laws, designed to restrain unauthorized sale of narcotics, are not adequate to control the evil, and that their enforcement has seemed impracticable.

Professor THEOPHILUS PARVIN, of Philadelphia, Pennsylvania, read a paper on the "Importance of Practical Obstetrics" in the course of instruction given by medical schools, in which he maintained that practical as well as theoretical obstetrics should be made a part of the regular course of every medical college, and asked the indorsement of the Academy in maintaining that position.

The annual address by the President of the Academy, Dr. F. H. GERRISH, of Portland, Maine, was delivered, in which he clearly demonstrated the necessity for more than a mere grammar-school education as proper preparation for the study of medicine, and presented the disadvantages under which the medical student labored without proper preliminary mental training before beginning his medical studies. He drew attention to the influence exerted by the State Board of Health of Illinois by establishing a minimum standard of requirements by medical colleges to entitle their diplomas to be recognized in the State of Illinois.

He further discussed certain amendments to the constitution of the Academy offered at previous meetings, one of which referred to the code of ethics. After consideration of the subject during the session of the Academy the matter was postponed until the next annual meeting.

Dr. R. L. SIBBET, of Carlisle, Pennsylvania, under the title of "A Few Words Concerning Our Academy," recalled its organization in 1876, and the fact that one of the objects of its establishment was to aid in elevating the standard of preliminary education among those intending to study medicine, and recalled the fact that at the time of its organization there were no State laws relating to the practice of medicine in our country that were rigidly enforced. He believed that the influence that had been brought to bear by the Academy through the Fellows resident in different sections of our country had done much to aid in the establishment of State boards of examiners which exclude from practice unqualified medical men, and that graded courses of instruction had been established in many of the best medical colleges in our country. He believed that the fact that the Academy had set a high standard for admission had exerted no inconsiderable influence in these advances made toward higher medical education in the last twelve years.

Dr. GEORGE J. FISHER, of Sing Sing, New York, read a paper entitled, "The Famous Historic Masters of the Healing Art were Men of Classical Education." In tracing the history of medicine from Hippocrates to the present time, he cited many instances in which medical men whose fame was most enduring were those who had had the advantages of liberal classical education with the mental discipline which such study implies, and urged that all practicable influence be brought to bear to secure the broadest and most liberal education for those contemplating the study of medicine.

Dr. L. D. BULKLEY, of New York City, read a paper on "The Relations Between the General Practitioner and the Consultant or Specialist," the object of it being to point out the mutual relations which should exist between the general practitioner and the specialist.

Dr. GHARES C. LEE, of New York City, read a paper entitled, "The Necessity for Post-graduation Instruction in the Present State of American Medical Education," citing the fact that the first post-graduate medical school was organized in this country in 1882; that several now are in existence in different cities in this country; that the advantages offered now for medical education no longer leave it essential for students of medicine to visit European medical schools, and that the chief advantage of the physician now visiting Europe is to enable him to institute comparisons in the methods of practice and of teaching, by familiarity with the methods of different countries, to liberalize his views.

Dr. HENRY I. BOWDITCH, of Boston, Massachusetts, read a paper entitled "Tolerance and Intolerance in Medicine; Codes of Ethics; What Code should this Academy Adopt," in which he repeated the views he entertains regarding the code of ethics of the American Medical Association, which have become well known to the medical profession.

He concluded by expressing the hope that the Academy, by the work of its individual members, by its own general labors, would tend to bring the whole profession of America up to higher grades of thought, or sentiment, and of action, so that we may at length really become what we have hitherto, but with unconscious falsehood, claimed to be, a truly liberal profession.

Dr. CHARLES McINTYRE, of Easton, Pennsylvania, read a paper whose title was "Which is the Liberal School?"

Dr. C. C. BOMBAUGH, of Baltimore, Maryland, read a paper on "The Multiplication of Useless Drugs," and referred to the great number indicated by the index of the fifteenth edition of the United States Dispensatory. He advocated materially curtailing the number to be embraced in the list of officinal preparations, and advocated determining more definitely the value of many of them by further experiments upon inferior animals.

A paper by Professor H. A. JOHNSON, of Chicago, Illinois, "Relative to the Influence of the Work of the Illinois Medical-Practice Act upon Medical Education," was read. (It appears elsewhere in this number of the *Journal and Examiner*.)

Two papers were read by title—one on "The Treatment of Uterine Disease by other than Surgical Means," by Professor W. F. WAUGH, of Philadelphia, Pennsylvania; the other, "The Evils of a Medical Dialect Separated Widely from Classical English," by Professor EDMUND ANDREWS, of Chicago, Illinois.

About one hundred new Fellows were elected. Those from Chicago were Drs. A. H. Foster, G. S. Isham, W. Van Hook, J. S. Knox, and E. B. Weston.

As honorary Fellows: Drs. J. H. Rauch, Springfield, Illinois; Sir Joseph Lister and Sir Spencer Wells, of London, England; J. L. Championière, Paris, Fiance; H. D. Didima, Syracuse, New York.

Officers elected for the ensuing year are:

President, Dr. Leartus Connor, Detroit, Michigan.

Vice-Presidents, Drs. P. D. Keyser, Philadelphia, Pennsylvania; L. D. Bulkley, New York City; T. Parvin, Philadelphia, Pennsylvania; G. J. Fisher, Sing Sing, New York.

Secretary and Treasurer, Dr. R. J. Dunglison, Philadelphia, Pennsylvania.

Assistant Secretary, Dr. Charles McIntyre, Jr., Easton Pennsylvania.

Place of next annual meeting, Chicago, Illinois.

